# Post-Transcriptional Mechanisms Respond Rapidly to Ecologically Relevant Thermal Fluctuations During Temperature-Dependent Sex Determination

**DOI:** 10.1093/iob/obaa033

**Published:** 2020-10-07

**Authors:** Samantha L Bock, Matthew D Hale, Faith M Leri, Philip M Wilkinson, Thomas R Rainwater, Benjamin B Parrott

**Affiliations:** 1 Odum School of Ecology, University of Georgia, Athens, GA 30602, USA; 2 Savannah River Ecology Laboratory, Aiken, SC 29802, USA; 3 Department of Biology, University of Virginia, Charlottesville, VA 22904, USA; 4 Department of Biology, University of Oklahoma, Norman, OK 73019, USA; 5 Tom Yawkey Wildlife Center, Georgetown, SC 29440, USA; 6 Belle W. Baruch Institute of Coastal Ecology & Forest Science, Clemson University, Georgetown, SC 29442, USA

## Abstract

An organism’s ability to integrate transient environmental cues experienced during development into molecular and physiological responses forms the basis for adaptive shifts in phenotypic trajectories. During temperature-dependent sex determination (TSD), thermal cues during discrete periods in development coordinate molecular changes that ultimately dictate sexual fate and contribute to patterns of inter- and intra-sexual variation. How these mechanisms interface with dynamic thermal environments in nature remain largely unknown. By deploying thermal loggers in wild nests of the American alligator (*Alligator mississippiensis*) over two consecutive breeding seasons, we observed that 80% of nests exhibit both male- and female-promoting thermal cues during the thermosensitive period, and of these nests, all exhibited both male- and female-promoting temperatures within the span of a single day. These observations raise a critical question—how are opposing environmental cues integrated into sexually dimorphic transcriptional programs across short temporal scales? To address this question, alligator embryos were exposed to fluctuating temperatures based on nest thermal profiles and sampled over the course of a daily thermal fluctuation. We examined the expression dynamics of upstream genes in the temperature-sensing pathway and find that post-transcriptional alternative splicing and transcript abundance of epigenetic modifier genes *JARID2* and *KDM6B* respond rapidly to thermal fluctuations while transcriptional changes of downstream effector genes, *SOX9* and *DMRT1*, occur on a delayed timescale. Our findings reveal how the basic mechanisms of TSD operate in an ecologically relevant context. We present a hypothetical hierarchical model based on our findings as well as previous studies, in which temperature-sensitive alternative splicing incrementally influences the epigenetic landscape to affect the transcriptional activity of key sex-determining genes.

## Introduction

Embryos often experience dynamic environmental conditions during development which can exert lasting influences on organismal phenotypes. This is especially true for species with temperature-dependent sex determination (TSD) in which the sex of offspring is determined by temperature cues experienced during discrete periods in development. Occurring in a diverse range of taxa, including all crocodilians, many turtles, and some fish (Bull 1980; Lang and Andrews 1994; Ospina-Alvarez and Piferrer 2008), TSD provides a unique window through which to examine how transient thermal cues coordinate molecular changes that ultimately establish patterns of inter- and intra-sexual variation. Major advances have recently been made in understanding the proximate developmental mechanisms underlying TSD (Czerwinski et al. 2016; Yatsu et al. 2016; Deveson et al. 2017; Ge et al. 2017, 2018), yet little is known regarding how these mechanisms interface with the dynamic thermal environments experienced by embryos in nature.

For many species with TSD, nest temperatures fluctuate considerably on daily, monthly, and seasonal timescales (Warner and Shine 2011; Escobedo-Galván et al. 2016; Carter et al. 2017; 2018). While the consequences of these temperature fluctuations for offspring phenotype are incompletely understood, in some species including the red eared slider turtle, *Trachemys scripta*, temperature fluctuations appear to play critical roles in influencing population sex ratios. Mean temperatures of *T. scripta* nests in the field rarely exceed those necessary to produce females under constant laboratory conditions, yet mark-recapture studies indicate females are frequently recruited into these populations. This apparent discordance is reconciled by the observation that female development can be triggered in *T. scripta* by as few as 5 days of exposure to female-promoting temperatures (FPTs; Carter et al. 2018). How do embryos integrate brief periods of FPT cues experienced in the field during “heatwaves” into lasting physiological responses that drive sexual fate commitment? And, how do these mechanisms function in other TSD species? These questions remain unanswered and hold important implications for our understanding of TSD as it occurs in ecologically relevant contexts.

Epigenetic mechanisms, including DNA methylation and histone modifications, appear to play upstream roles in integrating transient temperature cues into persistent transcriptional responses during TSD (Navarro-Martín et al. 2011; Matsumoto et al. 2013; Parrott et al. 2014; Ge et al. 2018). Two genes with roles in chromatin regulation, *JARID2* and *KDM6B* (or *JMJD3*), are among the earliest genes displaying temperature-sensitive differential expression during TSD in both *T. scripta* and *Alligator mississippiensis* (Czerwinski et al. 2016; Yatsu et al. 2016). *JARID2* is a component of the master Polycomb repressive complex 2 (PRC2) which plays a key role in localizing the PRC2 complex to its target loci for subsequent transcriptional silencing via histone 3 lysine 27 trimethylation (H3K27me3) (Peng et al. 2009; Landeira and Fisher 2011; da Rocha et al. 2014; Kaneko et al. 2014; Sanulli et al. 2015). *KDM6B* encodes a histone demethylase which removes the repressive H3K27me3 mark to activate transcription of target loci (Agger et al. 2007; Lan et al. 2007). Intriguingly, interruption of *KDM6B* activity via RNA interference in *T. scripta* results in the inhibition of male-development at a male-promoting temperature (MPT) and is associated with increased H3K27me3 at the promoter of *DMRT1*, a transcription factor involved in male development (Ge et al. 2018). Together, these findings suggest that epigenetic modifications and the conserved genes that regulate them play causal roles in integrating transient temperature cues experienced during development into sexually dimorphic transcriptional programs during TSD.

Interestingly, the role of these upstream epigenetic regulators in mediating TSD is likely more nuanced than previously realized. While much of the focus of TSD research has centered on investigating transcriptional responses to temperature, mounting evidence suggests post-transcriptional mechanisms, particularly alternative splicing, may contribute an added layer of regulatory complexity (Harry et al. 1990; Anand et al. 2008; Agrawal et al. 2009; Rhen et al. 2015; Deveson et al. 2017). Alternative splicing, the process by which pre-mRNA generates variable mature mRNA transcripts via differential exon usage and intron retention, plays a widespread role in contributing to transcriptional diversity across metazoan taxa (Tapial et al. 2017) and serves as the decisive sex-determining signal in some species (Salz 2011). Notably, multiple species possessing different forms of TSD including *A. mississippiensis*, *T. scripta*, and *Pogona vitticeps*, exhibit temperature dependent alternative splicing of the epigenetic modifier genes, *JARID2* and *KDM6B* (Deveson et al. 2017). In particular, a unique intron containing premature stop codons is differentially retained depending on incubation temperature in mature transcripts of each of these genes (Deveson et al. 2017). How environmental variability experienced in the field interfaces with these molecular pathways is currently unknown.

Here, we quantified the level of thermal variation experienced by alligator embryos in wild nests and implemented experimental thermal fluctuations based on empirically derived nest thermal profiles to examine how transcription and post-transcriptional alternative splicing of genes involved in sex determination and sexual differentiation change over the course of a daily thermal fluctuation during TSD. We found that alternative splicing of the epigenetic modifier *JARID2*, in both the gonad and brain, responded rapidly to thermal fluctuations. Further, transcript abundance of both *JARID2* and *KDM6B* also fluctuated with temperature in the gonad, albeit with an apparent delay of ∼7 h. In contrast to these epigenetic regulators, genes with downstream roles in sex determination and differentiation, *SOX9* and *DMRT1*, did not exhibit clear responses to thermal fluctuations. Together our findings suggest fluctuating thermal cues experienced in nature are integrated via a temporal hierarchy of molecular responses during TSD.

## Methods

### Field-derived nest thermal profiles

**Fig. 1:**
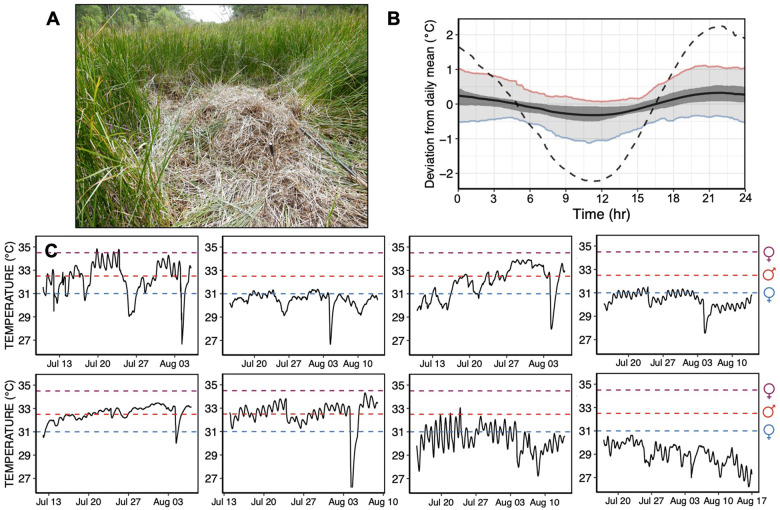
(**a**) Image of a representative alligator nest. (**B**) Design of experimental daily thermal fluctuation using the temperature deviation from the daily mean at 5-min increments in empirically derived nest thermal profiles. Solid black line depicts the average deviation from the mean daily nest temperature across all days in the TSP for eight nests monitored in 2015. Dark gray shading depicts the middle 50th percentile of deviations from the daily mean temperature and light gray shading depicts the middle 90th percentile of deviations from the daily mean temperature. Red and blue lines depict the 95th and 5th percentile of temperature deviations, respectively. Black dotted line depicts the deviation from the daily mean temperature of the experimental thermal fluctuation. Although the amplitude of the experimental trace is greater than the average thermal fluctuation observed in nature, the experimental trace is not outside of ecological relevance as natural nests are observed to fluctuate by 7°C within a single day. (**C**) Nest thermal profiles from nests monitored during 2015 on which the experimental thermal fluctuation was based. Horizontal dotted lines represent constant temperatures promoting female (blue, maroon) and male (red) development.

Twenty alligator nests were monitored at the Tom Yawkey Wildlife Center (YWC; Georgetown, SC; Bock et al. 2020). At each nest (Fig. 1A), one Onset (UTBI-001) HOBO temperature logger preprogrammed to record temperature at 5-min intervals was deployed in the center of the nest cavity and one temperature logger was attached to vegetation in close proximity to the nest out of direct sunlight to record air temperatures of the nest microclimate. Nests were left undisturbed until after hatchlings emerged (second week of September), after which temperature loggers were retrieved. Raw data from the temperature loggers were processed to only include measurements for the dates encompassing the thermosensitive period (TSP), Ferguson Stages 15–24 (Ferguson 1985; Lang and Andrews 1994; McCoy et al. 2015). Dates of the TSP were estimated based on hatch date, average nest temperature during the first 49 days of the incubation period, and the established relationship between temperature and developmental rate in *A. mississippiensis* (Kohno and Guillette 2013).

### Laboratory incubation experiment

Field collections were performed under permits obtained from the Florida Fish and Wildlife Conservation Commission and the US Fish and Wildlife Service. In 2018, three clutches of alligator eggs were collected shortly after oviposition at Lake Woodruff National Wildlife Refuge (De Leon Springs, FL). Following transport to the Savannah River Ecology Laboratory (Jackson, SC), a representative embryo was examined to determine the developmental stage of each clutch (Ferguson 1985). Eggs were kept in damp sphagnum moss and incubated at 32°C, a temperature promoting the development of both males and females, until the opening of the TSP at Ferguson Stage 15. Thermal sensitivity of sex determination does not begin until Stage 15 and embryos were kept at a single constant temperature prior to this stage to minimize variation resulting from other thermosensitive embryonic traits. Eggs were then shifted to one of the three temperature treatments—a constant 29°C (FPT), constant 33.5°C (MPT), or fluctuating thermal regime based on field-derived nest thermal profiles (average 31.25°C, minimum 29°C, maximum 33.5°C) (González et al. 2019). We derived the fluctuating thermal regime from the eight nest temperature profiles measured during 2015 by determining the average deviation from the mean nest temperature for every 5 min of the day during the TSP, then increasing that deviation by a factor of seven (Fig. 1B). This resulted in a thermal regime that fluctuated on a daily basis between the male-promoting (33.5°C) and female-promoting (29°C) temperatures and exhibited the same periodicity as a wild nest. While daily thermal variation of the experimental fluctuation was greater than average (average daily nest temperature shift = 0.84°C; experimental daily temperature shift = 4.5°C), it was not outside the scope of measured daily fluctuations (e.g., nests were observed to experience shifts of ≥7°C within a day).

**Fig. 2:**
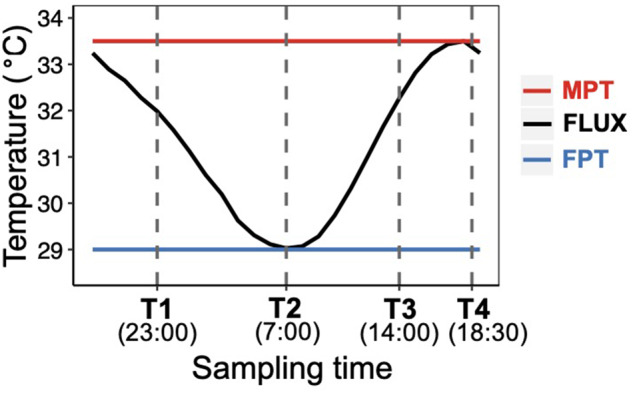
Experimental daily thermal regimes—constant MPT, fluctuating temperature, and constant FPT. Vertical dotted lines depict timepoints at which Stage 22 embryos were sampled.

At Stage 22, the middle of the TSP, alligator embryos was sampled at four time points spanning a daily thermal fluctuation in the fluctuating temperature treatment (FLUX ; Fig. 2). The sampling time points corresponded to the minimum temperature (29°C, FPT; T2; *n* = 10), intermediate increasing temperature (31.5°C, T3; *n* = 10), maximum temperature (33.5°C, MPT, T4; *n* = 9), and intermediate decreasing temperature (31.5°C, T1; *n* = 8) of the FLUX. Embryos exposed to a constant FPT (29°C; *n* = 11[T1], 10 [T2], 7 [T3], 8 [T4]), and constant MPT (33.5°C; *n* = 9 [T1], 8 [T2], 7 [T3], 11 [T4]) were sampled at the same time points to assess daily variability in gene expression unrelated to temperature and to yield a baseline to which the FLUX group could be compared.

### Nucleic acid isolation, cDNA synthesis, and qPCR

**Fig. 3:**
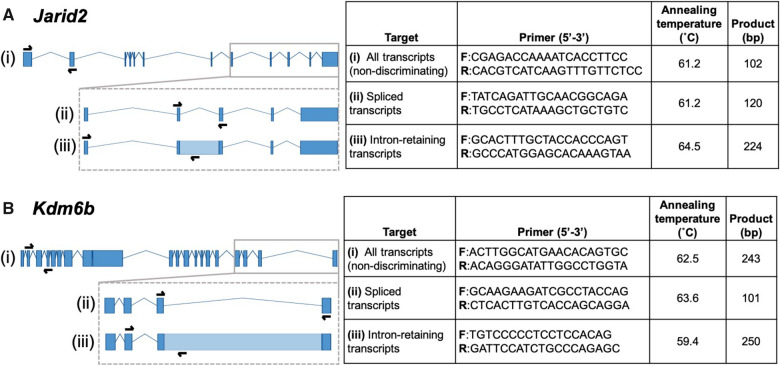
Gene isoforms with primer locations. (**A**) *JARID2* gene model for *A. mississippiensis*. (**B**) *KDM6B* gene model for *A. mississippiensis.* (i) Entire transcript with locations of primers selecting for all transcripts (not discriminating based on intron–retention status). (ii) Locations of primers selecting for intron-spliced transcripts. (iii) Locations of primers selecting for intron-retaining transcripts (11th intron in *JARID2* and 19th intron in *KDM6B*). Primer information is included in associated tables on right.

Gonadal and brain RNA were extracted using the SV total RNA isolation system (Promega; Madison, WI, USA) and resulting concentrations were quantified via spectrophotometry (NanoDrop One; Thermo Fisher Scientific; Waltham, MA, USA). Synthesis of cDNA was carried out using the iScript cDNA synthesis kit (Bio-Rad; Hercules, CA, USA) according to the manufacturer protocol and using 903.4 and 999 ng of input RNA from the gonad and brain, respectively. Gene expression was assessed via quantitative real-time PCR and reactions were performed in triplicate using an SYBR green reaction mix. Intron-retaining transcripts of *JARID2* and *KDM6B* were selectively targeted for quantification via specially designed primer sets. In particular, we targeted transcripts retaining the same introns reported to be differentially retained in previous studies (11th intron in *JARID2* and 19th intron in *KDM6B* [Deveson et al. 2017]). Information for all associated primers is provided in Fig. 3. All primers were designed to be intron-spanning. Those primers targeting intron-retaining isoforms were designed to span the preceding intron. For each gene and isoform, all expression values were derived from triplicate replicates (for biological and standard samples) using a standard curve comprised of target-containing plasmid standards of known concentration (copies/µL) that was run on each qPCR plate. Expression values for gonad and brain samples were normalized to that of beta-actin (*ACTB*) and ribosomal protein L8 (*RPL8*), respectively, which served as internal controls and were also derived from standard curves. There were no significant effects of temperature, sampling timepoint, or their interaction on the absolute expression levels of either of these housekeeping genes (Supplementary Fig. S2).

### Statistical analyses

Statistical analyses were conducted using R statistical software version 3.6.1. Distributions of relative expression values for each gene were tested for normality and homoscedasticity using a Shapiro–Wilk test and Levene’s test, respectively. If necessary, a transformation was applied to relative expression values to achieve normality. A log-transformation was applied to values for *JARID2* (all transcripts, intron–retention ratio), *KDM6B* (all transcripts, intron–retention ratio), and *SOX9*. A square-root transformation was applied to values for *DMRT1*. Transformations were not sufficient to alleviate non-normality for the *JARID2* intron–retention ratio in both the gonad and brain. We examined the influence of temperature treatment, sampling timepoint, and their interaction on relative expression of each gene and isoform using linear mixed effects models with clutch identity included as a random effect. These models were fit using the “lme4” package in R. We obtained *P*-values from the models using likelihood ratio tests. Due to the persistent non-normality of the *JARID2* intron–retention ratio following transformation, we also assessed the effect of temperature and sampling timepoint in the gonad and brain by conducting non-parametric Kruskal–Wallis tests. In both cases, results of the Kruskal–Wallis test conformed to those of the linear mixed effects model. To further examine potential relationships among the responses of genes in the gonad to thermal regime, we assessed pairwise Pearson correlations between each of the isoforms of *JARID2* and *KDM6B*, as well as *DMRT1* and *SOX9.*

## Results

### Nest thermal profiles

Thermal profiles of 20 alligator nest cavities were monitored between 2015 and 2016 at the YWC (Georgetown, SC, USA). The overall mean temperature during the TSP for all nests was 32.02°C, a temperature predicted to produce 69.8% males (Lang and Andrews 1994). The coolest nest monitored occurred in 2015 and had a mean nest temperature of 29.00°C, a temperature predicted to produce 100% females. The warmest nest monitored occurred in 2016 and had a mean nest temperature of 33.85°C, a temperature predicted to produce 51.9% males (Lang and Andrews 1994). We observed considerable within-nest variability in temperature (Fig. 1C), with 80% (16 of 20) of nests exhibiting both male- and FPT s within the TSP. All of these nests also exhibited both male- and FPTs within the span of a single day during at least one day during the TSP. On average, nest temperatures varied by 0.84°C within the span of a day. The maximum daily temperature range for an individual nest was 7.61°C and the minimum daily temperature range was 0.08°C. While individual nests varied considerably in their mean temperatures during the TSP, there were distinct commonalities in the shape of their daily thermal cycles (Supplementary Fig. S1). In particular, nests tended to exhibit their coolest temperatures between 06:00 and 18:00 h followed by a temperature maximum occurring between 18:00 and 24:00 h. Nests also tended to warm faster than they cooled (Supplementary Fig. S1). Each of these thermal characteristics was retained in our experimental thermal fluctuation wherein embryos experienced male- and FPTs over the course of a daily thermal cycle.

### Epigenetic modifiers

**Fig. 4:**
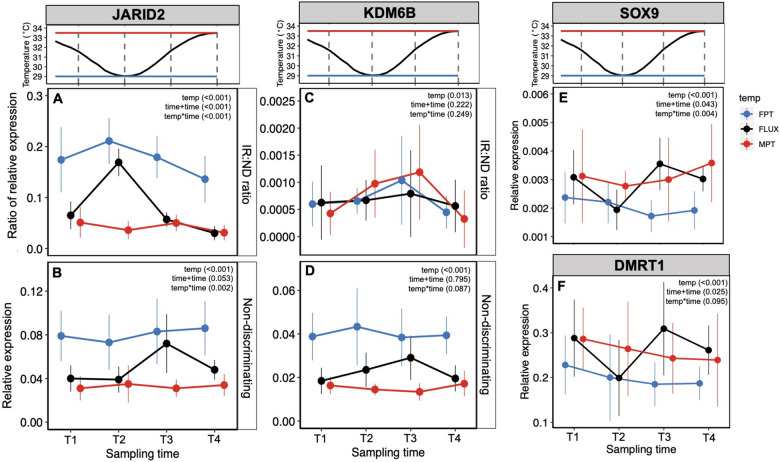
Gonadal gene expression of upstream epigenetic modifiers and downstream effectors of sex determination in response to different thermal regimes. (**A**) *JARID2* relative expression ratio of intron-retaining transcripts to all (nondiscriminating) transcripts (IR: ND) for each treatment across sampling time points. (**B**) *JARID2* relative expression of all transcripts. (**C**) *KDM6B* relative expression ratio of intron-retaining transcripts to all transcripts (IR: ND). (**D**) *KDM6B* relative expression of all transcripts. (**E**) *SOX9* relative expression. (**F**) *DMRT1* relative expression. All gene expression values normalized to the housekeeping gene *ACTB*. Central dots depict mean, vertical lines depict ±1SD.

In the gonad, there was a significant interactive effect of temperature treatment and sampling timepoint on the relative expression ratio of the *JARID2* intron-retaining transcripts to all transcripts (IR: ND; Χ^2^(6) = 49.86, *P* < 0.001; Fig. 4A). A Kruskal–Wallis test of the combined effect of temperature and timepoint on the *JARID2* intron–retention ratio supported this result (H [11] = 81.28, *P* < 0.001). While embryos exposed to the constant FPT exhibited consistently higher intron–retention in *JARID2* compared to embryos exposed to the constant MPT, embryos exposed to the FLUX exhibited variable levels of intron–retention. In particular, intron–retention of *JARID2* peaked in FLUX embryos at the timepoint in which these embryos were experiencing a FPT (T2), consistent with a rapid response of intron–retention to temperature (Fig. 4A). We also detected a significant interactive effect of temperature treatment and sampling timepoint on overall transcript abundance of *JARID2* (non-discriminating; Χ^2^ [6] = 20.64, *P* = 0.002; Fig. 4B). Similar to the pattern of intron–retention in *JARID2*, transcript abundance of *JARID2* was more variable in FLUX embryos. However, in contrast to intron–retention, transcript abundance of *JARID2* peaked at the timepoint after they experienced a FPT (T3; ∼7 h after T2; Fig. 4B).

**Fig. 5:**
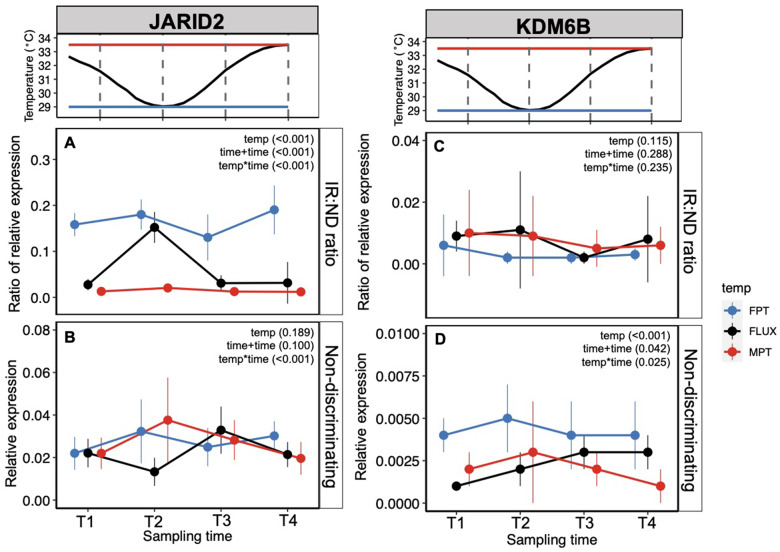
Brain gene expression of upstream epigenetic modifiers in response to different thermal regimes. (**A**) *JARID2* relative expression ratio of intron-retaining transcripts to all transcripts (IR: ND) for each treatment across sampling time points. (**B**) *JARID2* relative expression of all transcripts. (**C**) *KDM6B* relative expression ratio of intron-retaining transcripts to all transcripts (IR: ND). (**D**) *KDM6B* relative expression of all transcripts. All gene expression values normalized to the housekeeping gene *RPL8*. Central dots depict mean, vertical lines depict ±1SD.

In the brain, intron–retention in *JARID2* exhibited a nearly identical response to temperature as it did in the gonad suggesting a tissue-independent mechanism. There was a significant interactive effect of temperature treatment and sampling timepoint on *JARID2* intron–retention in the brain (IR: ND; Χ^2^ [6] = 34.105, *P* < 0.001; Fig. 5A), with intron–retention peaking in FLUX embryos at the FPT timepoint, T2. This result was also supported by a Kruskal–Wallis test of the combined effect of temperature and timepoint on *JARID2* intron–retention (H [11] = 83.70, *P*< 0.001). The response of *JARID2* transcript abundance to temperature in the brain was less clear, but there was a significant interactive effect of temperature treatment and sampling timepoint (non-discriminating; Χ^2^ [6] =  28.954, *P* < 0.001; Fig. 5B).

Levels of intron–retention in *KDM6B* were lower than those of *JARID2* in both the gonad and brain. There was a significant effect of temperature treatment (Χ^2^ [2] = 8.6665, *P* = 0.013) but not sampling timepoint or their interaction on the ratio of *KDM6B* intron-retaining transcripts to all transcripts in the gonad (Fig. 5C). There was also a significant effect of temperature treatment (Χ^2^ [2]  = 84.346, *P* < 0.001) but not sampling timepoint or their interaction on *KDM6B* transcript abundance in the gonad (Fig. 5D). While gonadal *KDM6B* transcript abundance appeared highest in the FLUX embryos at the timepoint after embryos received an FPT cue (T3), this variation was not statistically significant. In contrast to *JARID2*, there was no significant effect of the temperature-by-timepoint interaction on *KDM6B* transcript abundance (Fig. 5D). In the brain, intron–retention levels in *KDM6B* were low and there were no effects of temperature, sampling timepoint, or their interaction on the ratio of intron-retaining to all *KDM6B* transcripts. We did, however, detect a significant interactive effect of temperature and timepoint on *KDM6B* transcript abundance in the brain (Χ^2^ [6]  = 14.478, *P* = 0.025) (Fig. 5D).

### Downstream effector genes of sex determination

There was a significant interactive effect of temperature and timepoint on gonadal *SOX9* relative expression (Χ^2^ [6] = 19.2, *P* = 0.004). Consistent with its role in promoting testicular differentiation, *SOX9* relative expression was higher in embryos incubated at MPT compared to those incubated at FPT (Fig. 4E). FLUX embryos exhibited variation in their relative expression of *SOX9* with highest relative expression observed at timepoint T3 (one timestep after experiencing an FPT or three timesteps after experiencing an MPT; Fig. 4E). Given the current experimental design, it is not yet possible to distinguish whether this reflects a rapid response of *SOX9* relative expression to increasing temperatures following exposure to FPT or a delayed response to MPT.

We observed a similar pattern of relative expression of *DMRT1* compared to that of *SOX9*, though we did not detect a significant interactive effect of temperature and timepoint on *DMRT1* relative expression. There was, however, a significant additive effect of temperature and timepoint (Χ^2^ [3] = 9.3691, *P* = 0.025) on *DMRT1* relative expression in the gonad. Similar to *SOX9*, relative expression of *DMRT1* in FLUX embryos was highest at timepoint T3 (one timestep after experiencing an FPT or three timesteps after experiencing an MPT; Fig. 4F), though this did not result in a significant temperature-by-timepoint interactive effect.

The strongest gene expression correlations we observed in the gonad were between the intron-retaining and non-discriminating isoforms for *JARID2* and *KDM6B*, the non-discriminating isoform for *JARID2* and the non-discriminating isoform for *KDM6B*, and the intron-retaining isoform for *JARID2* and non-discriminating isoform for *KDM6B* (Supplementary Fig. S3). While we observed a correlation between *DMRT1* and *SOX9* expression, correlations between expression of upstream epigenetic regulators, *KDM6B* and *SOX9*, and downstream effector genes, *DMRT1* and *SOX9*, were comparatively weaker (Supplementary Fig. S3).

## Discussion

**Fig. 6:**
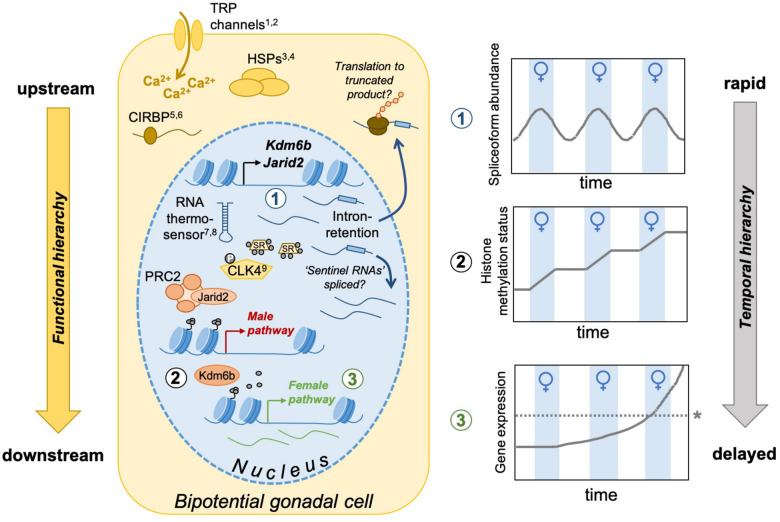
Hypothesized model linking functional and temporal hierarchies of molecular responses to fluctuating thermal cues during TSD. Temperature is initially transduced into a biological signal via one or more thermo-sensory mechanisms. Possible mechanisms include TRP channels which alter intracellular calcium levels, HSPs, CIRBP, RNA thermosensors, or CLK4 which phosphorylate serine arginine-rich proteins with roles in alternative splicing. Upstream epigenetic regulators, *JARID2* and *KDM6B*, are proximate targets of this cellular thermo-sensory mechanism and their transcription and post-transcriptional regulation respond rapidly to thermal fluctuations as a result (1) In particular, transcript abundance of *JARID2* and *KDM6B* increases in the bipotential gonad following exposure to transient female-promoting thermal signals. Intron–retention in *JARID2* also responds rapidly to temperature potentially leading to the generation of a truncated protein with altered function or sequestration of “sentinel RNAs” in the nucleus that are later spliced and translated. Once translated, JARID2 and KDM6B influence H3K27me3 marks at the promoters of genes associated with the male and female pathway resulting in incremental changes to the chromatin landscape (2) in response to temperature. Over time, the repressive H3K27me3 mark accumulates at the promoters of genes involved in the male-pathway via the actions of JARID2 in complex with PRC2, while genes involved in the female-pathway progressively lose H3K27me3 at their promoters via actions of *KDM6B*. As a result, expression of genes involved in the female-pathway increases gradually (3) until reaching a threshold (asterisks) for sexual fate commitment. ( References: ^1^Lin et al. 2018; ^2^Yatsu et al. 2015; ^3^Bentley et al. 2017; ^4^Kohno et al. 2010; ^5^Rhen and Schroeder 2010; ^6^Schroeder et al. 2016; ^7^Chowdhury et al. 2006; ^8^Johansson et al. 2002; ^9^Haltenhof et al. 2020 ).

During TSD, alligator embryos experience rapid fluctuations between FPT and MPT in wild nests. Findings from this study suggest embryos integrate these opposing environmental signals via a temporal hierarchy of responses. Alternative splicing of the epigenetic modifier gene *JARID2* responds quickly to thermal fluctuations during sex determination in both the gonad and brain. Transcript abundance of both *JARID2* and *KDM6B* similarly fluctuates with temperature, though with an apparent time delay relative to the splicing response. In contrast to these upstream epigenetic regulators, expression patterns of downstream effector genes of sex determination, *DMRT1* and *SOX9*, are highly variable and do not exhibit as clear a relationship to temperature under fluctuating conditions. These results are consistent with a hypothetical model wherein alternative splicing and transcript abundance of upstream chromatin modifiers fluctuate with temperature resulting in incremental epigenetic changes that influence the transcriptional activity of key sex determining genes (Fig. 6). However, additional experiments testing other key components of this conceptual model, such as how temperature-dependent alternative splicing affects the function of *KDM6B* and *JARID2*, are clearly still needed.

The relatively rapid responses of *JARID2* and *KDM6B* to thermal fluctuations suggest these factors are likely proximate targets of a cellular thermo-sensory mechanism(s) and thus occupy upstream positions in the sex-determining transcriptional cascade. Despite extensive research efforts, the thermo-sensory mechanism governing TSD has remained elusive. Several candidates have been hypothesized to initially translate incubation temperature into a biological response during TSD including transient receptor potential channels (Yatsu et al. 2015; Lin et al. 2018), heat shock proteins (Kohno et al. 2010; Bentley et al. 2017; ), and cold-inducible binding protein (CIRBP) (Rhen and Schroeder 2010; Chojnowski and Braun 2012; Schroeder et al. 2016; Lin et al. 2018) (Fig. 6). All of these factors are generally well-conserved and coordinate cellular responses to temperature across diverse systems (Clapham 2003; Kohno et al. 2010; Zhong and Huang 2017) though evidence for their role in TSD is currently equivocal. CIRBP represents an intriguing candidate as it belongs to a family of RNA recognition motif binding proteins with roles in regulating splicing, translation, and mRNA stability (Dreyfuss et al. 2002; De Leeuw et al. 2007; Xia et al. 2012). Based on data from *Chelydra serpentina*, temperature-induced transcriptional responses of *CIRBP* would appear to lag behind the shifts in alternative splicing and transcript abundance of *JARID2* and *KDM6B* reported here (Schroeder et al. 2016). This, however, does not rule out the possibility of rapid post-transcriptional regulation of CIRBP by temperature (Gotic et al. 2016; Haltenhof et al. 2020). A recent report demonstrated a role for CDC-like kinases in regulating temperature-sensitive alternative splicing of both *CIRBP* and *JARID2* (Haltenhof et al. 2020), lending support to the possibility that multiple thermo-sensory mechanisms may act in a coordinated manner during TSD. Further investigations into temperature-induced changes in intracellular signaling cascades (Rottingen and Iversen 2000; Stamm 2008), protein conformation (Haltenhof et al. 2020), and/or conformation of RNA molecules themselves (Johansson et al. 2002; Chowdhury et al. 2006; Loh et al. 2013) during TSD are likely to shed light on the molecular interactions that transduce temperature into a sex-determining signal.

Exposure to FPTs in alligator embryos resulted in both increased intron–retention and increased overall transcript abundance of *JARID2*. Counterintuitively, the intronic sequence retained in mature *JARID2* transcripts contains premature stop codons and intron-retaining transcripts are predicted to produce truncated products with altered or ameliorated function if they avoid nonsense-mediated decay and are translated (Deveson et al. 2017). This result is consistent with previous findings in which intron–retention in both *JARID2* and *KDM6B* was more frequent at the temperature promoting expression of these genes in *A. mississippiensis* and *T. scripta* (Deveson et al. 2017; Georges and Holleley 2018). Various explanations have been posited to reconcile these seemingly incongruent observations. For example, intron–retention in *JARID2* and *KDM6B* may influence which genes these factors target for transcriptional regulation or alter how these factors interact with other chromatin modifier complexes, though these ideas have yet to be tested experimentally (Wong et al. 2016; Georges and Holleley 2018; Marasca et al. 2018). Our finding that intron–retention and transcript abundance respond to fluctuating temperatures on different timescales may provide additional insight into the consequence of this temperature-sensitive alternative splicing in ecologically relevant contexts. It is possible that rapid intron–retention in *JARID2* stabilizes the female-promoting signal during transient decreases in nest temperature during alligator TSD by promoting the storage of intron-retaining transcripts in the nucleus which are later spliced and transcribed to functional proteins after the FPT cue. An analogous phenomenon is observed in a fern species, *Marsilea vestita*, and sea anemone, *Nematostella vectensis* (Moran et al. 2008; Boothby et al. 2013; Wong et al. 2016) in which intron-retaining transcripts are stored in the nucleus and serve as “sentinel RNAs” that are later spliced facilitating rapid stage-specific protein translation. Testing whether a similar process plays a role in TSD would necessitate further experiments to determine if intron-retaining transcripts are eventually spliced and translated to functional products (e.g., experiments employing pulse-chase methods). Alternatively, intron–retention serves widespread roles in gene regulation and repression of protein translation (Wong et al. 2013, 2016; Braunschweig et al. 2014), and may serve to buffer against premature sexual fate commitment resulting from temperature-induced transcription of *JARID2* and *KDM6B*, thereby extending the period of temperature-sensitivity during TSD. Previous work suggests intron–retention can take on diverse functions across taxa, especially depending on the genomic context of the retained introns (Wong et al. 2016), and thus further experiments to resolve the function of intron–retention in chromatin-modifier genes are likely to yield important insights into TSD.

Many genes with conserved roles in sex determination and differentiation display robust sexually dimorphic expression patterns in TSD species after the TSP (Czerwinski et al. 2016; Yatsu et al. 2016). Yet in this study, we detect highly variable expression patterns of two of these genes, *SOX9* and *DMRT1*, in the middle of the TSP, especially in embryos exposed to a thermal fluctuation. This finding raises the question—how do sexually dimorphic transcriptional patterns arise during TSD and how does this relate to the regulatory actions of *JARID2* and *KDM6B* over time? Ge et al. demonstrated that in *T. scripta* under constant MPTs, *KDM6B* promotes commitment to the testicular fate via direct removal of the repressive H3K27me3 mark at the promoter of *DMRT1* (Ge et al. 2018). However, under fluctuating conditions in alligator embryos, *JARID2* and *KDM6B* transcript abundance fluctuates with temperature, while downstream effector genes exhibit apparent delays in their responses. This raises the possibility that under fluctuating thermal conditions, chromatin landscapes resulting from the actions of *JARID2* and *KDM6B* may be dynamically remodeled over time to eventually repress or activate target downstream effector genes. However, it remains unknown whether incremental epigenetic changes occur during TSD under fluctuating conditions, and if so, what ultimately regulates the threshold for sexual fate commitment (Fig. 6). Further, the full battery of genes targeted by *JARID2* and *KDM6B* is yet to be resolved in most TSD species, including *A. mississippiensis.*

While this study revealed intriguing patterns in the responses of upstream epigenetic regulators and downstream effector genes of sex determination to a thermal fluctuation between MPT and FPTs, there are limitations to the current experimental design that leave several important questions unanswered. In particular, while transcript abundance of *JARID2* and *KDM6B* reached a maximum at the timestep after receiving a FPT cue (∼7-h delay), this observation is, in part, a result of our sampling scheme and may not reflect the true timescale of this response. In order to better resolve the temporal scale on which alternative splicing, transcription, and translation respond to fluctuating thermal cues during TSD, future experiments must sample embryos across a higher resolution time-series and across multiple days. Furthermore, the thermal regime implemented here represents a simplification of the thermal fluctuations occurring in wild nests. Nests frequently exhibit thermal fluctuations of smaller magnitudes and daily fluctuations often vary over the course of the TSP. It remains unknown how characteristics such as the magnitude and repeatability of fluctuations impact molecular responses to these thermal cues and, ultimately, how these complex thermal profiles shape sex ratio outcomes in species with TSD.

Many questions regarding the epigenetic mechanisms of TSD await further exploration and studies like the one presented here underscore the importance of implementing thermal regimes that accurately reflect the dynamic environment in which TSD evolved and examining these processes across multiple temporal scales in future experimental investigations of this phenomenon.

## Supplementary Material

obaa033_Supplementary_Data
