# Supplementary figures and images for: Post-Transcriptional Mechanisms Respond Rapidly to Ecologically Relevant Thermal Fluctuations During Temperature-Dependent Sex Determination
Source: Integr Org Biol. 2020 Oct 7;2(1):obaa033. doi: 10.1093/iob/obaa033 (PMC7715621; doi:10.1093/iob/obaa033)

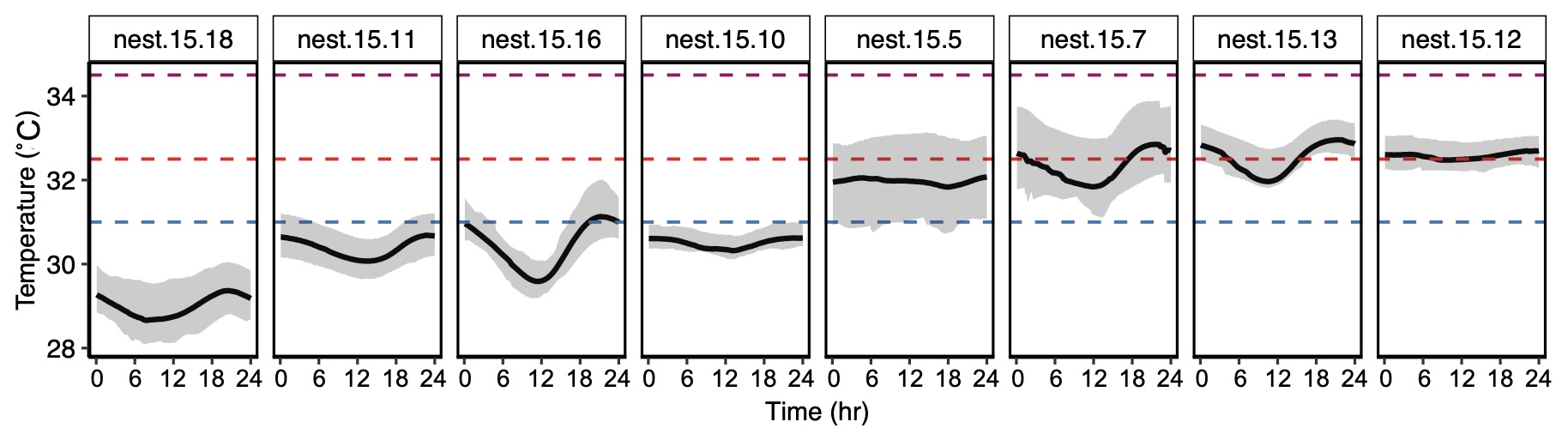

Supplement: obaa033_Supplementary_Data [file obaa033_supplementary_data.zip › SI_FIGURE1_FINAL.jpg]

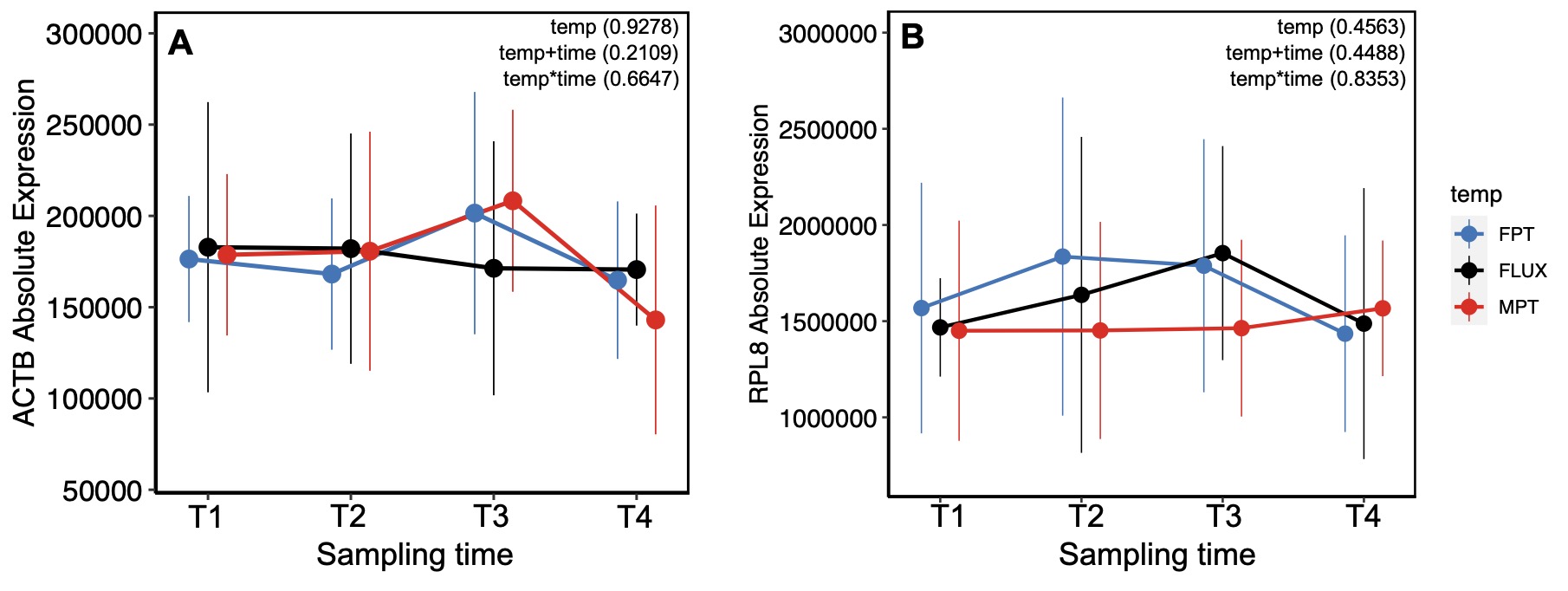

Supplement: obaa033_Supplementary_Data [file obaa033_supplementary_data.zip › SI_FIGURE2_FINAL.jpg]

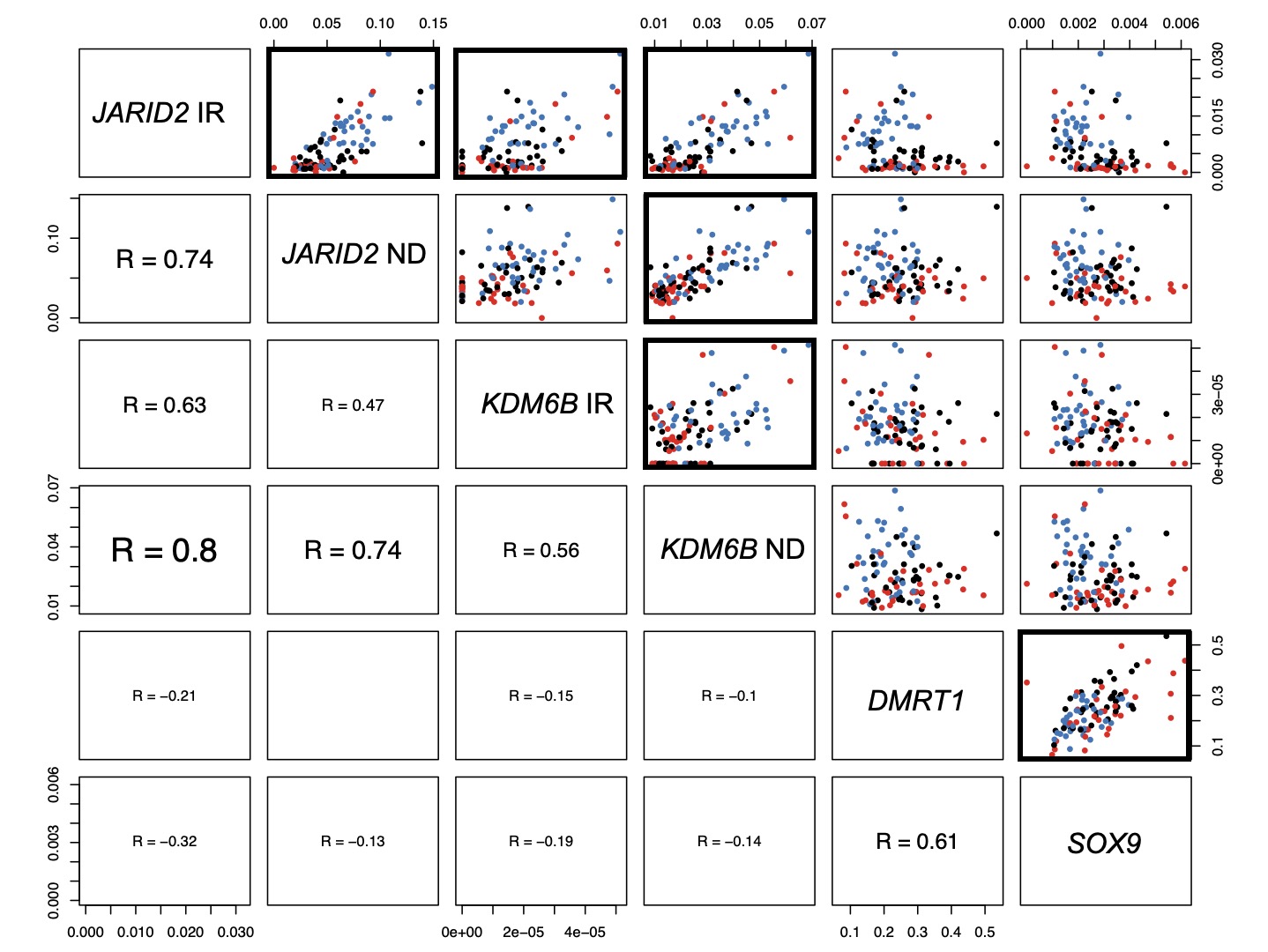

Supplement: obaa033_Supplementary_Data [file obaa033_supplementary_data.zip › SI_FIGURE3_FINAL.jpg]
